# Healthcare Fragmentation and Cardiovascular Risk Control Among Older Cancer Survivors in the REasons for Geographic and Racial differences in Stroke (REGARDS) Study

**DOI:** 10.1007/s11764-020-00933-4

**Published:** 2020-09-08

**Authors:** Laura C. Pinheiro, Evgeniya Reshetnyak, Monika M. Safford, David Nanus, Lisa M. Kern

**Affiliations:** 1 Division of General Internal Medicine, Department of Medicine, Weill Cornell Medicine, New York, NY; 2 Division of Hematology and Oncology, Department of Medicine, Weill Cornell Medicine, New York, NY

**Keywords:** cancer survivors, CVD risk factor control, care fragmentation, diabetes, self-rated health

## Abstract

**Purpose:**

Cardiovascular disease (CVD) is the number one cause of death among 5-year cancer survivors. Survivors see many providers and poor coordination may contribute to worse CVD risk factor control. We sought to determine associations between fragmentation and CVD risk factor control among survivors overall and by self-rated health.

**Methods:**

We included REGARDS participants aged 66+ years who: 1) had a cancer history; 2) reported diabetes, hypertension or hyperlipidemia; and 3) had continuous Medicare coverage. Twelve month ambulatory care fragmentation was calculated using the Bice-Boxerman Index (BBI). We determined associations between fragmentation and CVD risk factors, defining “control” as fasting glucose <126 mg/dL or non-fasting glucose <200 mg/dL for diabetes; blood pressure <140/90 mm Hg for hypertension; and total cholesterol <240 mg/dL, low-density lipoprotein cholesterol <160 mg/dL, or high-density lipoprotein cholesterol >40 mg/dL for hyperlipidemia.

**Results:**

The 1,002 cancer survivors (2+ years since cancer treatment) had mean age of 75 years, 39% were women, and 23% were Black. Among individuals with diabetes (N=225), hypertension (N=660), and hyperlipidemia (N=516), separately, approximately 60% had CVD risk factor control. Overall, more fragmented care was not associated with worse control. However, among cancer survivors with excellent, very good or good health, more fragmentation was associated with a decreased likelihood of diabetes control (OR 0.78, 95% CI 0.61–0.99), adjusting for confounders.

**Conclusions:**

More fragmented care was associated with worse glycemic control among cancer survivors with diabetes who reported excellent, very good, or good health. Associations were not observed for control of hypertension or hyperlipidemia.

**Implications for cancer survivors:**

Reducing fragmentation may support glucose control among survivors with diabetes.

## Introduction

The number of cancer survivors[1] is expected to grow by 25% over the next decade, with 20 million survivors anticipated by 2026 [2]. Cardiovascular disease (CVD) is the #1 non-cancer cause of death among individuals who survive 5 or more years from their cancer diagnosis [3–7]. There are several reasons why CVD is the leading cause of death. First, there is evidence that some cancer treatments have direct cardiotoxic effects [8–13]. Second, CVD is common in the general population (92 million Americans with CVD) [14] and risk of CVD increases considerably with age [15]. However, a third possible reason is that cancer survivors may receive sub-optimal care for their CVD risk factors, compared to individuals without cancer [16, 17]. This may be because their care focuses primarily on surveillance for cancer recurrence, or because the providers they see are not typically focused on CVD risk management [16, 17]. For example, previous studies suggest that survivors who see exclusively oncologists are more likely to receive cancer-related screenings, but less likely to receive non-cancer preventive care (e.g., flu vaccines, eye exams for diabetics, cholesterol screening) [17]. As such, cancer survivors may be at increased risk for poor CVD outcomes.

Following a cancer diagnosis, cancer survivors often see many different healthcare providers for their cancer and non-cancer care [18]. In fact, a recent study found that cancer survivors have significantly more fragmented care (i.e., care from multiple providers without a dominant provider) even 2 or more years after the conclusion of their active cancer treatments, compared to a comparison group without a history of cancer [19]. While these care patterns may be appropriate, fragmented care may increase the risk of potential gaps in communication across healthcare providers.^[20]^ More fragmentation has been associated with repeated testing and an increased risk of hospitalization, compared to less fragmentation [21–23].

We are unaware of any studies that have examined the relationship between fragmentation and CVD risk factor control among cancer survivors. Cancer registry datasets such as SEER-Medicare are often used to examine health service use and health outcomes among cancer survivors, but SEER-Medicare relies on claims data and does not include lifestyle factors (smoking, exercise, diet) or physiologic assessments such as blood pressure, cholesterol, and glucose. Our study addresses these limitations by using data from the REasons for Geographic And Racial Differences in Stroke (REGARDS) cohort study, a racially diverse, population-based, longitudinal dataset linked to fee-for-service Medicare claims [24] to determine, among cancer survivors 66 years and older, if more fragmented ambulatory care is associated with worse CVD risk factor control, compared to less fragmented ambulatory care. Determining if fragmentation is associated with CVD risk factor control in cancer survivors is critical to modifying risks that lead to worse CVD outcomes among cancer survivors. That is, our findings could be used to inform management strategies or future interventions to improve CVD risk factor management and control among cancer survivors.

## Methods

### REGARDS Cohort Study:

REGARDS is a national, prospective cohort study studying racial and geographic disparities in stroke outcomes. Between 2003–2007, REGARDS recruited 30,239 community dwelling, English-speaking individuals ≥45 years of age and continues to follow participants for 10+ years [24]. At enrollment, a 45-minute computer assisted telephone interview (CATI) collected participants’ demographic data and medical history. At baseline, all participants underwent an in-home visit, which included a physical exam, medication inventory, and lab tests. All participants provided written informed consent. This study was approved by the Institutional Review Board at the University of Alabama at Birmingham and Weill Cornell Medical College. All participants provided written informed consent.

### Medicare claims:

As previously described, among adults who gave consent, REGARDS was linked to Medicare fee-for-service claims [25].

### Study Design:

Our study used baseline REGARDS data from 2005–2007 and Medicare fee-for-service claims for the 12 months before the baseline survey (2004–2007).

### Study Cohort:

There were 20,403 REGARDS participants linked to Medicare. Among those individuals, we included adults who: 1) were ≥66 years old at baseline, 2) had continuous Medicare fee-for-service coverage for 365 days before their baseline survey, 3) did not have end-stage renal disease, 4) had a self-reported history of cancer, and 5) had ≥4 ambulatory visits in the 12 months before baseline, because calculating fragmentation scores using <4 visits leads to unstable statistical estimates [26]. We determined a participant’s history of cancer using two self-reported questions on the baseline survey, “Have you ever been diagnosed with cancer?” and “Have you been treated with chemotherapy or radiation in the past two years?”. If someone answered “yes” to both questions they were not eligible to enroll in the REGARDS study. If the individual answered “yes” to the first question and “no” to the second question they were eligible. Our study was limited to individuals with a self-reported history of cancer.

We identified three separate groups of interest among the cancer survivors: those with self-reported diabetes, hypertension, and/or hyperlipidemia. Participants were considered to have each of these conditions if they self-reported them in the baseline REGARDS survey. Among those who self-reported one of these conditions, information regarding use of anti-glycemic medication or insulin (for diabetes), antihypertensive medication (for hypertension), and cholesterol-lowering medications (for hyperlipidemia) was collected.

### Key Independent Variable:

The Bice-Boxerman Index (BBI) was used to calculate Medicare claims-based healthcare fragmentation in the 12 months prior to the baseline REGARDS survey [27–29]. This index captures care “dispersion” (spread of a patient’s care across multiple providers) and “density” (relative share of visits by each provider) [30]. Prior studies found the BBI to be highly correlated with other measures of fragmentation [31]. To calculate the BBI, participants’ ambulatory visits, the number of unique ambulatory providers they saw, and the number of visits to each provider in a 12-month period of Medicare claims were considered. We defined ambulatory visits using a modified definition from the National Committee for Quality Assurance [32]. Applied modifications limited the visit definition to office-based evaluation and management visits for adults in the outpatient setting [33]. Possible BBI score values range from 0 to 1 with 0 indicating low continuity (or high fragmentation) and 1 indicating high continuity (or low fragmentation). For ease of interpretation, we reversed the BBI scoring so that higher scores would reflect more fragmentation [33, 34]. Our analyses use the reversed Bice-Boxerman Index (rBBI).

### Study Outcome:

The three primary study outcomes were control of diabetes (among those with diabetes), control of hypertension (among those with hypertension), and control of hyperlipidemia (among those with hyperlipidemia). “Good control” was defined as: 1) fasting glucose <126 mg/dL or non-fasting glucose <200 mg/dL for diabetes; 2) blood pressure <140/90 mmHg for hypertension; and 3) total cholesterol <240 mg/dL, LDL<160 mg/dL or HDL >40 mg/dL for hyperlipidemia. These definitions were based on guidelines used in 2005–2007 (when individuals completed the baseline surveys) from the American Diabetes Association [35], the Joint Committee on Prevention, Detection, Evaluation and Treatment of High Blood Pressure [36], and The National Cholesterol Education Program [37].

### Potential Confounders:

Guided by Andersen’s Behavioral Model of Health Services Use [38], we selected covariates that fell into three categories: predisposing characteristics, enabling resources, and evaluated need. Together, these factors influence health service use. Predisposing characteristics included demographic factors such as a participant’s age at baseline, race (Black or White), and gender. Enabling resources included socioeconomic factors such as having a low annual household income (<$35,000/year) or low educational attainment (<high school), residing in a rural region (rural urban commuting area [RUCA] codes 9 and 10) or the Southeast stroke-belt region (belt/buckle, defined as North Carolina, South Carolina, Georgia, Tennessee, Mississippi, Alabama, Louisiana and Arkansas; or non-stroke belt), living in a health professional shortage area (HPSA), living in a zip code with high poverty, and living in one of the 9 US states with the least public health infrastructure [39]). Perceived need was represented by an individual’s self-rated general health status using the Short-Form-1 (SF-1). Evaluated need included the Charlson Comorbidity Index [40] to reflect comorbidity burden.

### Statistical Analyses:

Cohort characteristics were described for cancer survivors overall. As we suspected that fragmentation may vary by self-rated health based on past reports [41], an interaction between rBBI and self-rated health (SF-1) was tested. Given the statistical significance of the interaction term, subsequent analyses were stratified by SF-1- groups. Given sample size constraints, individuals with excellent, very good, and good health were grouped together and compared to those who reported fair and poor health. Characteristics between survivors with excellent, very good, or good health were compared to those with fair or poor health. For unadjusted comparisons, we used t-tests for continuous variables that were normally distributed, Wilcoxon rank-sum test for skewed continuous variables, and chi-square tests for categorical variables. Examining Medicare fee-for-service claims in the 365 days before the baseline survey, we measured ambulatory care patterns (number of visits, number of providers, percent of visits with the most frequently seen provider, and rBBI) overall and compared patterns between individuals with excellent, very good. or good health and those with fair or poor health using Wilcoxon tests.

We described the proportion of individuals who had diabetes, hypertension, and hyperlipidemia control at baseline, separately, allowing participants to contribute to more than one group. We then assessed the relationship between fragmentation and CVD risk factor control, overall and stratified by self-rated health groups. Then, we selected potential confounders that were significantly associated with the CVD risk factor control in bivariate models (p<0.05). Using these covariates, we estimated multivariable logistic regression models for the adjusted association between rBBI and CVD risk factor control for each risk factor, separately. We explored interaction terms between rBBI and gender as well as rBBI and race for each risk factor, separately. Consistent with our prior work, individuals with a rBBI equal to 0.00 and 1.00 were dropped from our adjusted models, as this is a small, heterogenous group of individuals [19]. We conducted sensitivity analyses that included individuals with rBBI of 0.00 and 1.00 and compared results to our primary results.

We implemented multiple imputation of missing covariates [42]. Annual household income was the covariate with the highest proportion of missingness (13%). All other covariates had less than 10% missing. We used multivariable imputation by chained equations (MICE) and used classification and regression trees (CART) as an imputation engine because it captures potential non-linear effects [43, 44]. We obtained 20 imputed data sets with 20 iterations, and the results were merged using Rubin’s rules. Data imputation procedures were performed in R version 3.4.1 “mice” package. All other analyses were conducted in SAS Version 9.4 with 2-sided statistical tests and significance levels of 5%.

## Results

### Cancer Survivor Characteristics:

**Figure 1.**
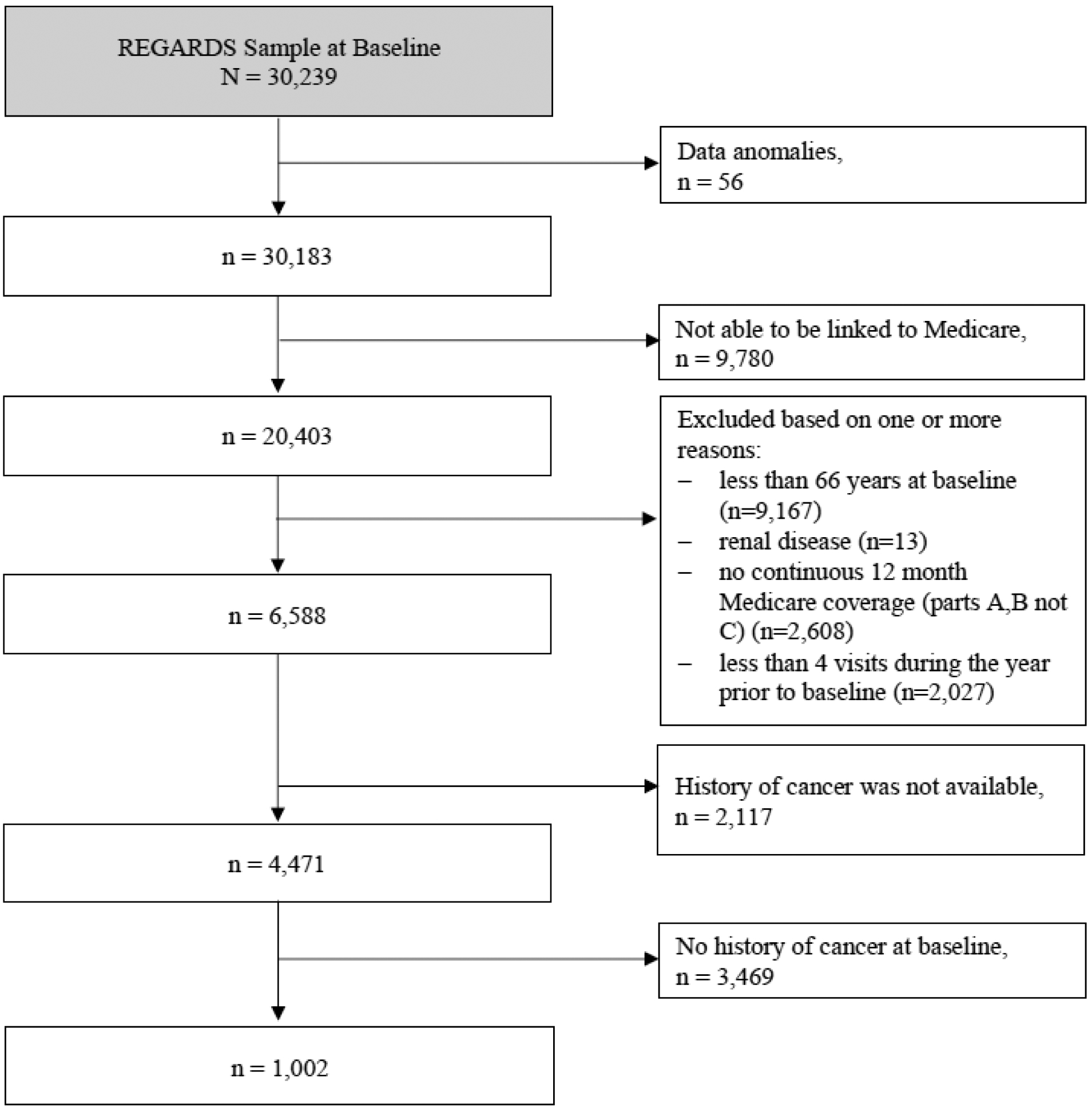
Exclusion Cascade

1,002 REGARDS participants met eligibility criteria and had a self-reported history of cancer (Figure 1). The mean age at baseline was 75 years, 39% were women, and 23% were Black (Table 1). Overall, 789 (79%) reported excellent, very good or good health, and 210 (21.0%) report fair or poor health. In the 12 months before the baseline survey, cancer survivors had a median of 10 visits (IQR 7–15) with 5 providers (rBBI 0.80) and 43% of visits were with their most frequently seen provider (Table 2). Cohort characteristics by self-rated health groups are shown in Supplementary Table 1.

### Diabetes Control:

Among the 225 cancer survivors with diabetes, 179 (80%) reported taking anti-glycemic medication or insulin at baseline. Of the 225 cancer survivors with diabetes, 61% had their diabetes controlled at baseline. Overall, there was no statistically significant association between more fragmented care and diabetes control (OR 0.97; 95% CI 0.84–1.13, p=0.73). However, there was a statistically significant interaction between rBBI and SF-1 (p=0.01). The association between fragmentation and diabetes control varied between individuals with poor/fair health versus those with excellent, very good, and good health. Among individuals with excellent, very good or good health, a 0.1 unit increase in rBBI was significantly associated with a (aOR 0.78; 95% CI 0.61–0.99) decreased odds of having diabetes control after adjusting for potential confounders (Table 4). No significant association between rBBI and diabetes control was observed for individuals with poor/fair health. Interaction terms between rBBI and gender and rBBI and race, separately, were not statistically significant (p=0.16 and p=0.61, respectively).

### Hypertension Control:

Among the 660 cancer survivors with hypertension, 59% had control at baseline. Of the 660 participants, 617 (94%) reported taking an anti-hypertensive medication. We did not observe a statistically association between healthcare fragmentation and hypertension control (OR 1.07; 95% CI 0.97–1.17, p=0.19). The interaction term between rBBI and the SF-1 was similarly not significant (p=0.25), nor was the interaction terms between rBBI and gender and rBBI and race (p=0.14 and p=0.09, respectively).

### Hyperlipidemia Control:

Among the 516 cancer survivors with hyperlipidemia, 60% had control at baseline and 352 (68%) reported taking a cholesterol-lowering medication. We did not observe a statistically significant association between healthcare fragmentation and hyperlipidemia control (OR 1.11; 95% CI 0.99–1.23, p=0.07). The interaction term between rBBI and the SF-1 was also not significant (p=0.24), and the interaction terms between rBBI and gender and rBBI and race was also not, significant (p=0.47 and p=0.56, respectively).

## Discussion

Among Medicare beneficiaries in REGARDS who were 66+ years old, nearly 40% of cancer survivors with diabetes did not have controlled glucose, hypertension or hyperlipidemia. We found that more fragmentation of ambulatory care was associated with worse glycemic control among cancer survivors with diabetes who self-rated excellent, very good or good health, but not among those with fair or poor health. This association was not observed for hypertension or lipid control overall or by self-rated health.

Previous studies have shown that compared to non-cancer controls, cancer survivors are less likely to receive CVD risk factor management services, in particular those related to diabetes care [45]. This is concerning because cancer survivors are known to have higher prevalence of CVD risk factors (e.g., diabetes and hypertension) compared to age-matched non-cancer controls [46]. However, unlike our study that examined lab values to operationalize glucose control, prior work among cancer survivors has considered only receipt of lab testing such as HbA1c and LDL tests. As such, our findings make an important contribution to the cancer survivorship literature by quantifying the relationship between fragmented care and glucose control. Until now, little has been known about patterns of ambulatory care and CVD risk factor control outside of cancer registries [46] and clinical trials, which have limited generalizability to usual care. Our study generates evidence that suggests there may be a link between increased fragmentation and worse glucose control among a national sample of cancer survivors. Given that diabetes is a known CVD risk factor and that CVD is the leading cause of death among cancer survivors, increasing control of diabetes among cancer survivors is a promising opportunity to improve CVD outcomes in this population.

To our knowledge, our study is the first to establish an association between more fragmented care and worse diabetes control among cancer survivors with diabetes. This link is important because it identifies a modifiable factor (i.e., fragmented care) that, if found to be causally related, could be reduced and potentially help to improve diabetes control among cancer survivors. Some fragmentation may be driven by medical need, but a previous study identified numerous causes of fragmentation that were not related to medical need and instead were related to provider-level, practice-level, or environment-level factors [20]. Future studies could explore whether at least some of the fragmentation of care among cancer survivors is modifiable (that is, not driven by medical need). Then interventions to decrease unnecessary fragmentation among cancer survivors with diabetes and good self-related health could be developed and tested. Decreasing unnecessary fragmentation would consolidate care among the most medically needed providers. This would likely allow better communication across those providers, a goal consistent with a recent recommendation from the American College of Cardiology [47].

### Limitations:

Our study has some notable limitations. First, cancer status was determined by self-report on the REGARDS baseline survey and we do not have detailed data on the type of cancer or treatments received. Second, beyond the two-year minimum required for enrollment in REGARDS, we do not know how long participants were free from cancer. Third, we defined diabetes control using glucose values but recognize that HbA1c values would give us a more robust measurement of glycemic control; however, these lab values were not available in our dataset. Finally, CVD risk factor control was assessed at a single time point and does not reflect fluctuations in blood pressure, cholesterol, and glucose over time.

## Conclusions

We found that, among cancer survivors with excellent, very good, or good self-rated health (which was nearly 80% of cancer survivors), having more fragmented ambulatory care was associated with worse glycemic control than less fragmented care. Future studies should explore the association between fragmentation, CVD risk factor control (in particular diabetes), and CVD outcomes. Understanding the mechanisms by which fragmentation affects CVD risk factor control among cancer survivors with good self-related health will enable the design of future interventions to decrease unnecessary fragmentation among these individuals.

## Supplementary Material

11764_2020_933_MOESM1_ESM

## Figures and Tables

**Table 1:** Cohort Characteristics

Factor	Level	Cancer Survivors
N		1,002
Age, mean (SD)		74.9 (5.9)
rBBI, median (IQR)^a^		0.80 (0.67, 0.86)
Black, N (%)		234 (23.4%)
Female, N (%)		389 (38.8%)
Low annual household income (<$35,000), N (%)		503 (50.2%)
Low educational attainment (<High school), N (%)		135 (13.5%)
Rural residence, N (%)^b^		18 (1.8%)
Southeast region residence (Stroke Belt/Buckle), N (%)^c^		546 (54.5%)
SF-12 physical component score, median (IQR)^d^		47.41 (37.5, 53.3)
SF-12 mental component score, median (IQR)^e^		57.39 (53.5, 59.9)
Residence in the Health Professionals Shortage Area (HPSA), N (%)		387 (38.6%)
Residence in the state with poor public health infrastructure, N (%)^f^		391 (39.0%)
Residence in a zip code with > 25% residents living below Federal poverty line, N (%)		172 (17.2%)
SF-1, self-rated health, N (%)	Excellent/Very Good/Good	789 (78.7%)
	Fair / Poor	210 (21.0%)
Charlson/Deyo comorbidities index, N (%)^g^	0	457 (45.6%)
	1	289 (28.8%)
	2	148 (14.8%)
	3	105 (10.5%)

a Reversed Bice-Boxerman Index (rBBI) calculated as 1-BBI, where larger scores correspond to higher fragmentation

b Rural urban commuting area [RUCA] codes 9 and 10

c REGARDS study oversampled residents from the stroke belt (Alabama, Arkansas, Louisiana, Mississippi, Tennessee, and the noncoastal regions in North Carolina, South Carolina, and Georgia) and the stroke buckle (the coastal regions within North Carolina, South Carolina, and Georgia).

d Ranges from 0 to 100, and a higher score indicates better physical health.

e Ranges from 0 to 100, and a higher score indicates better mental health.

f Public Health Infrastructure is calculated based on the America’s Health Ranking data; nine states (Louisiana, New Mexico, Mississippi, Nevada, South Carolina, Florida, Arkansas, Texas, Tennessee) fell into the bottom 20% of the US states with the worst public health infrastructure for at least 8 years during the ten-year period prior REGARDS enrollment (1993–2002).

g The index is the sum of the scores for each of the comorbid conditions weighted by its severity

**Table 2:** Overall Distribution of Fragmentation

	N	Mean	Median	P25	P75	Std
rBBI	1,002	0.75	0.80	0.67	0.86	0.17
# of visits	1,002	11.96	10.00	7.00	15.00	7.64
# of providers	1,002	5.02	5.00	3.00	6.00	2.38
% of visits with the most frequently seen providerr	1,002	0.45	0.43	0.33	0.56	0.17

a Reversed Bice-Boxerman Index (rBBI) calculated as 1-BBI, where larger scores correspond to higher fragmentation

**Table 3:** Distribution of Fragmentation Among Individuals with Diabetes by SF-1

	Total sample	SF-1 (Poor/Fair)	SF-1 (Good/Very good, Excellent)	p-value (between SF groups)
N	223	74	148	
rBBI, Median (IQR)^a^	0.8 (0.6, 0.8)	0.8 (0.6, 0.9)	0.8 (0.6, 0.8)	0.39
# of visits, Median (IQR)	12.0 (8.0, 17.0)	14.5 (10.0, 20.0)	11.0 (7.0, 14.0)	<0.001
# of providers, Median (IQR)	5.0 (3.0, 7.0)	6.0 (4.0, 7.0)	4.0 (3.0, 6.0)	<0.001
% of visits with the most frequently seen provider, Median (IQR)	0.5 (0.3, 0.6)	0.4 (0.3, 0.6)	0.5 (0.4, 0.6)	0.18

a Reversed Bice-Boxerman Index (rBBI) calculated as 1-BBI, where larger scores correspond to higher fragmentation

**Table 4:** Associations Between Healthcare Fragmentation and Diabetes Control Among Individuals with Diabetes and Cancer

	Effect	Adjusted OR (95% CI)	p-value
Model 1 (complete case, crude)	rBBI	0.97 (0.84, 1.13)	0.73
Model 2 (complete case, adjusted for SF-1 and rBBI)	rBBI at SF-1 (fair/poor)	1.34 (1.01, 1.79)	0.01*
	rBBI at SF-1 (good/very good, excellent)	0.82 (0.67, 1.02)	
Model 3 (complete case, fully adjusted)^a^	rBBI at SF-1 (fair/poor)	1.19 (0.83, 1.71)	0.01*
	rBBI at SF-1 (good/very good, excellent)	0.65 (0.47, 0.89)	
Model 4 (multiple imputed, fully adjusted)^a^	rBBI at SF-1 (fair/poor)	1.34 (0.95, 1.89)	0.01*
	rBBI at SF-1 (good/very good, excellent)	0.78 (0.61, 0.99)	

a Fully adjusted models with all covariates from Table 1.

* Statistical significance, p<0.05
