# Supplementary material for: Healthcare Fragmentation and Cardiovascular Risk Control Among Older Cancer Survivors in the REasons for Geographic and Racial differences in Stroke (REGARDS) Study
Source: J Cancer Surviv. Author manuscript; Available in PMC 2022 Apr 1. (PMC7937763; doi:10.1007/s11764-020-00933-4)
Supplement: 11764_2020_933_MOESM1_ESM [file NIHMS1627260-supplement-11764_2020_933_MOESM1_ESM.docx]

**Supplementary Materials.**

Supplementary Table 1. Cohort characteristics by SF-1 groups

| **Factor** | **Level** | SF-1  (Poor/Fair) | SF-1  (Good/Very good/Excellent) | *p*-value |  |
| --- | --- | --- | --- | --- | --- |
| N |  | 210 | 789 |  |  |
| Age, mean (SD) |  | 74.8 (5.6) | 74.9 (5.9) | 0.94 |  |
| rbbi, median (IQR) |  | 0.77 (0.62, 0.85) | 0.80 (0.69, 0.86) | 0.02 |  |
| Black, N (%) |  | 78 (37.1%) | 155 (19.7%) | <.0001 |  |
| Female, N (%) |  | 93 (44.3%) | 295 (37.4%) | 0.07 |  |
| Low income, N (%) |  | 134 (63.8%) | 368 (46.6%) | <.0001 |  |
| Lack of education, N (%) |  | 51 (24.3%) | 81 (10.2%) | <.0001 |  |
| Rural, N (%) |  | 5 (2.7%) | 13 (1.6%) | 0.47 |  |
| SE region, N (%) |  | 124 (59.1%) | 420 (53.2%) | 0.13 |  |
| PCS, median (IQR) |  | 32.4 (26.4, 42.2) | 49.7 (42.5, 54.6) | <.0001 |  |
| MCS, median (IQR) |  | 55.2 (48.7, 59.5) | 57.8 (54.5, 60.2) | <.0001 |  |
| HPSA, N (%) |  | 77 (36.7%) | 309 (39.2%) | 0.51 |  |
| Public health infrastructure, N (%) |  | 85 (40.5%) | 304 (38.5%) | 0.61 |  |
| Zip poverty, N (%) |  | 43 (20.5%) | 129 (16.3%) | 0.15 |  |
| Charlson Deyo index, N (%) | 0 | 53 (25.2%) | 404 (51.3%) | <.0001 |  |
|  | 1 | 58 (27.6%) | 231 (29.3%) |  |  |
|  | 2 | 46 (21.9%) | 100 (12.7%) |  |  |
|  | 3 | 51 (24.3%) | 53 (6.7%) |  |  |
